# Combined contribution of biochar and introduced AM fungi on lead stability and microbial community in polluted agricultural soil

**DOI:** 10.3389/fmicb.2023.1284321

**Published:** 2023-11-15

**Authors:** Xuedong Chen, Lin Tang, Kongyang Wu, Yifan Mo, Qian Tang, Gaojie Li, Ying Zhu

**Affiliations:** ^1^College of Life Science, Luoyang Normal University, Luoyang, Henan, China; ^2^School of Physics and Engineering, Henan University of Science and Technology, Luoyang, Henan, China

**Keywords:** Pb pollution, arbuscular mycorrhiza fungi, biochar, microbial community, farmland soil

## Abstract

**Introduction:**

Lead (Pb) pollution in agricultural soil has been accelerated by industrial development and human activities, and poses a major threat to agricultural ecosystems. Both biochar and arbuscular mycorrhiza (AM) fungi are considered to play an important role in remediation of Pb contaminated soil.

**Methods:**

The combined remediation effects of introduced AM fungi and biochar on soil properties, Pb availability, microbial community and functional profiles were systematically investigated in unsterilized Pb-polluted agricultural soil.

**Results:**

Results indicated that soil nutrients were significantly improved through the combined application of biochar and introduced AM fungi. The introduced AM fungi combined with biochar prepared at 400°C and 500°C promoted the transformation of Pb to a more stable state with low bioavailability. Moreover, the addition of AM fungi and biochar affected the relative abundances of dominant bacteria and fungi at the phylum and genus levels. Biochar mainly affected soil bacterial community and obviously increased the relative abundance of Actinobacteria and *Blastococcus*. The interactions between biochar and introduced AM fungi mainly affected fungal community, and increased the abundance of Ascomycota and *Botryotrichum*. Further, PICRUSt analysis indicated biochar amendment supported stronger bacterial metabolic functional potentials.

**Discussion:**

Therefore, the combined application of biochar and Therefore, the combined application of biochar and introduced AM fungi could improve soil nutrients, reduce Pb introduced AM fungi could improve soil nutrients, reduce Pb availability, availability, and show and show a positive effect on a positive effect on indigenous microbial communities and indigenous microbial communities and metabolic functions in metabolic functions in farmland soil.

## 1. Introduction

With the increase of human industrial and agricultural activities, lead (Pb) pollution in agricultural soils is a growing environmental issue of global concern. Pb is not biodegradable and tends to have long-lasting adverse effects on the soil environment through acute and diffuse pollution (Harindintwali et al., 2020). The microenvironment of agricultural soil is related to carbon, nitrogen, phosphorus, potassium, and other nutrient cycle processes, which should be paid more attention in the process of Pb remediation. However, there are still many problems in the remediation of Pb pollution in agricultural soil, such as high cost, long cycle, and great difficulty (Hou et al., 2020). Therefore, it is necessary to develop safe, efficient, and reproducible remediation technologies from single to joint.

Biochar is a solid product obtained by pyrolysis of residues of waste biomass produced in agriculture or forestry. It has developed pore structure, rich oxygen-containing functional groups, large specific surface area, high pH value, and cation exchange capacity (Xiang et al., 2022; Zhang and Guan, 2022). Thus, it can adsorb metal ions in soil and effectively reduce the available state of Pb in soil. Pb can be divided into five forms: exchangeable state, acid extractable state, reducible state, oxidizable state, and residual state. The exchangeable state and acid extractable state of Pb in soil have strong mobility, are easy to be absorbed and utilized by plants, and have great potential environmental effects. The reducible state and oxidizable state can be transformed into acid extractable state under certain conditions, which can be indirectly absorbed and utilized by plants. Residual heavy metals are mainly found in the soil lattice, with high stability, which are not easily released in a short time and cannot be absorbed and utilized by plants (Wang et al., 2005, 2022). Bian et al. (2014) found that biochar addition considerably reduced Pb bioavailability by 59.1%. Cui et al. (2016) also found that biochar could decrease Pb bioavailability by transforming the exchangeable fractions of Pb into relatively stable fractions (residual and organic). But not all forms of biochar are environmentally friendly or beneficial, because the properties and sorption capacity of biochar are affected by the type of raw material and the thermochemical conversion process used to produce the biochar, temperature, time, and heating rate, etc. (Wang et al., 2021).

Microbial remediation is also considered to be a cost-effective and eco-friendly method for the remediation of soil Pb pollution. Arbuscular mycorrhiza (AM) fungi are a kind of important soil microorganism, which can form symbiotic relationships with about 80% of terrestrial plants. AM fungi can not only help plants absorb water and mineral nutrients, but also help plants resist various biotic and abiotic stresses, such as heavy metals, saline alkali, drought, etc., playing an important role in agricultural ecosystems (Lehmann et al., 2014). AM fungi can absorb and accumulate Pb by fungal cell wall and intra-radical and extra-radical mycelium through the mechanism of chelation, surface precipitation, and ion exchange (Riaz et al., 2021). Moreover, AM fungi in association with plants can secrete glomalin related soil protein. As a stubborn, viscous and hydrophobic soil protein, glomalin has a remarkable potential in chelating Pb ions and reducing their bioavailability through metal speciation in soil (Jia et al., 2016; Wang et al., 2019; Stefanowicz et al., 2020).

Individual biochar or AM fungi has its own limitations when used separately to remediate contaminated soils (Xiang et al., 2022), necessitating comprehensive research of their combined use for Pb remediation. In recent years, more and more attention has been paid to the synergistic effects of biochar and AM fungi, focusing mainly on the promotion effects of the combined application of biochar and AM fungi on soil nutrients and physiological characteristics such as plant biomass, photosynthetic characteristics, element uptake, antioxidant activity and mitigation of heavy metal phytotoxicity in heavy metal polluted soils (Zhang et al., 2019; Gujre et al., 2021; Turan, 2021). However, little attention has been paid to the changes of native microbial communities and functions in soil during the Pb remediation process.

Biochar can affect soil microbial activity and biomass, and even reshape the structure of microbial community by providing a habitat and soil nutrients for microorganisms, changing the environmental conditions (air, water content and pH) of microbial habitat, and interfering interspecific or intraspecific communication of microbial cells (Xiang et al., 2022). AM fungi can also directly change rhizosphere microenvironment and microbial diversity (Feng et al., 2023; Zhao et al., 2023). Therefore, we hypothesized that the joint application of biochar and AM fungi could change the community and function of soil microorganism in the remediation process of Pb-contaminated soil. In addition, under field conditions, most soils contain diverse populations of indigenous AM fungi, and a sterilized soil situation is rare in nature. So in order to explore more practical remediation effects, the present study aimed to evaluate the effects of combined application of introduced AM fungi and biochar on soil Pb speciation and indigenous microbial communities and functions in unsterilized polluted farmland soil.

## 2. Materials and methods

### 2.1. Experimental materials

Based on preliminary investigation, a polluted maize field around a Pb smelter was selected in Henan Province. The surface soil (0–20 cm) in the maize field was collected and transported back to the laboratory. After the removal of plant debris, the soil was air-dried and sieved through a 5-mm mesh screen for use. The pH of the collected soil was 7.2, and the total Pb content was 1169.70 mg/kg.

The biochar was prepared by pyrolyzing corn straws, respectively at 300°C, 400°C, and 500°C in a nitrogen gas atmosphere. The specific surface area and pore size of the biochar were determined by a specific surface and pore size analyzer (V-Sorb2800P, Beijing), and the surface morphology characteristics of the biochar were characterized by a scanning electron microscope (Phenom, Netherlands).

The AM fungi species *Rhizophagus intraradices*, which was propagated and preserved in the laboratory, was selected for use in pot experiments.

### 2.2. Experimental design

The pot experiment designed the following 8 treatments: no introduced AM inoculation or biochar addition (NM0), no introduced AM inoculation but 300°C biochar addition (NM3), no introduced AM inoculation but 400°C biochar addition (NM4), no introduced AM inoculation but 500°C biochar addition (NM5), introduced AM inoculation and 300°C biochar addition (AM3), introduced AM inoculation and 400°C biochar addition (AM4), introduced AM inoculation and 500°C biochar addition (AM5), introduced AM inoculation but without biochar addition (AM0). Each treatment was repeated eight times. An aliquot of raw soil (~1.0 kg) was first thoroughly mixed with biochar at a dose of 3% and then placed into a plastic pot. The AM fungi inoculum was added with 30 g per pot, and 30 g of sterilized inoculum was also added for treatment without inoculation. The disinfected and germinated maize seeds were sown in the pot. All pots were randomized at regular intervals to compensate for differences in light and temperature in the greenhouse.

### 2.3. Soil properties

After the seedlings were harvested, the physical–chemical properties of air-dried soil samples were determined according to Bao (2000). pH was measured using a 1:2.5 soil/solution ratio in distilled water. The organic matter was measured using the potassium dichromate volumetric method. The alkali-hydrolyzable N was measured by using the alkaline hydrolysis nitrogen diffusion method. The available P was quantified using the molybdenum antimony colorimetric method. The available K was determined using CH_3_COONH_4_ extraction flame spectrophotometry.

### 2.4. Total Pb content and Pb speciation

The air-dried soil samples screened by 0.15 mm were digested by aqua regia-perchloric acid and concentrated nitric acid at high temperature, and the total Pb content in the soil was determined by flame atomic absorption spectrometry. The method developed by Tessier et al. (1979) was used to fractionate the Pb content into exchangeable, acid extractable, reducible and oxidizable fractions. The extracts were analyzed for Pb using inductively coupled plasma -atomic emission spectrometry (ICP-AES).

### 2.5. AM fungal colonization

5 g frozen roots was cleared in 10% KOH at 90°C for 30 min, bleached in alkaline H_2_O_2_ for 20 min, acidified in 1% HCl for 3 min, and stained in lactophenol trypan blue, and finally discolored with a 50%(v/v) glycerol solution (Phillips and Hayman, 1970). The colonization level was estimated using the gridline intersect method described by Giovannetti and Mosse (1980).

### 2.6. Soil DNA extraction and sequencing

Total genomic DNA samples were extracted from fresh soil samples using the OMEGA Soil DNA Kit (M5636-02) (Omega Bio-Tek, Norcross, GA, USA), following the manufacturer’s instructions, and stored at −20°C prior to further analysis. The quantity and quality of DNA extracted were measured using a NanoDrop NC2000 spectrophotometer (Thermo Fisher Scientific, Waltham, MA, USA) and agarose gel electrophoresis, respectively. PCR amplification of the bacterial 16S rRNA genes V3–V4 region was performed using the forward primer 338F (5′-ACTCCTA CGGGAGGCAGCA-3′) and the reverse primer 806R (5′-GGAC TACHVGGGTWTCTAAT-3′). The fungal ITS1 region gene was amplified using primers ITS5-1737F (5′-GGAAGTAAAAGTCGTA ACAAGG-3′) and ITS2-2043R (3′-GCTGCGTTCTTCATCGA TGC-5′). Then, the sequencing of rRNA genes was conducted on the Illumina HiSeq platform in Shanghai Personal Biotechnology Co., Ltd. The sequence data generated in this study were deposited in the NCBI Sequence Read Archive (SRA) and are available under the BioProject ID: PRJNA893452 and PRJNA893455 for bacteria and fungi, respectively.

### 2.7. Analysis for bacterial and fungal community composition

Microbiome bioinformatics were performed with QIIME2 2019.4 (Bolyen et al., 2018) with slight modification according to the official tutorials.^1^ Briefly, raw sequence data were demultiplexed using the demux plugin followed by primers cutting with cutadapt plugin (Martin, 2011). Sequences were then quality filtered, denoised, merged and chimera removed using the DADA2 plugin (Callahan et al., 2016). Non-singleton amplicon sequence variants (ASVs) were aligned with mafft (Katoh et al., 2002) and used to construct a phylogeny with fasttree2 (Price et al., 2009). Alpha-diversity metrics and beta diversity metrics were estimated using the diversity plugin with samples were rarefied. Taxonomy was assigned to ASVs using the classify-sklearn naïve Bayes taxonomy classifier in feature-classifier plugin (Bokulich et al., 2018) against the SILVA Database (Release 132) for bacteria and UNITE Database (Release 8.0) for fungi (Koljalg et al., 2013).

### 2.8. Prediction of bacterial function

The functional traits of the bacterial communities were predicted using PICRUSt based on the KEGG pathway database (Langille et al., 2013). We compared bacterial functional profiles at KEGG modules levels 2. Since fungal genome data is currently limited, the PICRUSt analyses were performed only for bacterial communities.

### 2.9. Statistical analysis

The differences in soil properties, Pb concentration and proportion of Pb fractions, and microbial diversity and abundance among different treatments were compared using two-way analysis of variance (ANOVA) at a significance level of 0.05. Pearson correlation analysis was performed to explore the relationship of soil properties with microbial diversity. Two-way ANOVA and Pearson correlation analysis were performed by IBM SPSS 20.0 software (SPSS Inc., Chicago, IL, USA). The composition of bacterial and fungal communities were compared by principal coordinates analysis (PCoA) complemented with an adonis permutational multivariate analysis of variance based on the Bray-Curtis distance distances. All the above data were visualized using the Origin 9.0 software or R package “ggplot2.” Redundancy analysis (RDA) was performed to explore the relationships between the soil chemical properties and microbial communities in Canoco 4.5. For the RDA model, each RDA axis and explanatory variables were tested using a Monte Carlo permutation test in Canoco.

## 3. Results

### 3.1. Characteristics of biochar prepared at different temperatures

The biochar prepared at 500°C had a comparatively higher pH and ash content than that prepared at 300°C or 400°C. The specific surface area of biochar prepared at 300°C was the smallest, followed by that prepared at 500°C, and the specific surface area of biochar prepared at 400°C was the largest, which was 6.88 m^2^/g (Table 1). The biochar prepared at 300°C had a large pore size, but small pore size was rare, so the specific surface area was small. The biochar prepared at 400°C and 500°C had many pores and a large specific surface area, especially the biochar prepared at 400°C had a good adsorption capacity (Supplementary Figures S1, S2).

**Table 1:** Basic properties of maize straw biochar prepared at different pyrolysis temperatures.

Biochar	Specific surface area (m^2^/g)	Ash content (%)	pH	Carbon (%)	Nitrogen (%)
BC300	1.13	32.36	7.54	42.52	2.07
BC400	6.88	32.46	8.65	40.68	1.47
BC500	4.83	53.54	9.47	42.84	1.49

BC300, biochar prepared at 300°C; BC400, biochar prepared at 400°C; BC500, biochar prepared at 500°C.

### 3.2. AM fungal colonization

**Figure 1:**
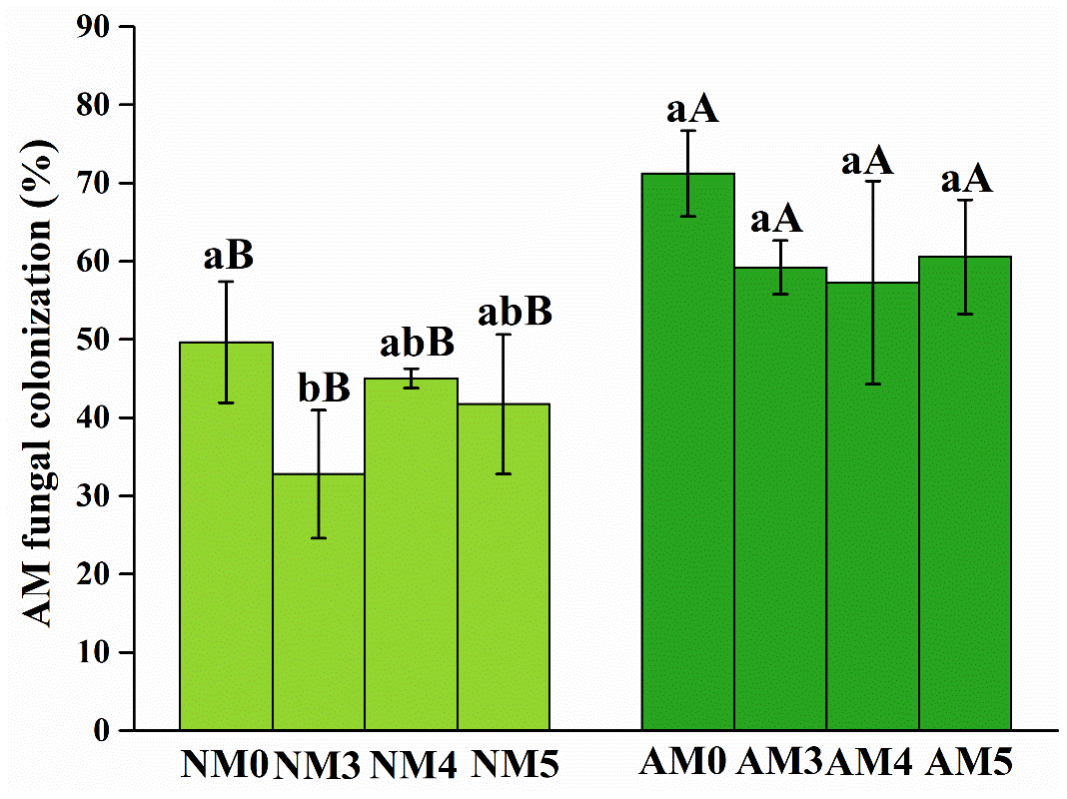
AM fungal colonization of maize roots under different treatments. NM0, no AM inoculation or biochar addition; NM3, no AM inoculation but 300°C biochar addition; NM4, no AM inoculation but 400°C biochar addition; NM5, no AM inoculation but 500°C biochar addition; AM3, introduced AM inoculation and 300°C biochar addition; AM4, introduced AM inoculation and 400°C biochar addition; AM5, introduced AM inoculation and 500°C biochar addition; AM0, introduced AM inoculation and no biochar addition. Different lower-case letters indicate significant differences among biochar treatments (Duncan’s comparisons, *p* < 0.05) and upper-case letters indicate significant differences between AM fungal inoculation treatments (NM vs. AM, Tukey’s comparisons, *p* < 0.05).

The roots of all maize seedlings were well colonized by AM fungi. The inoculation of introduced AM fungi significantly increased the root colonization rates under AM fungi and biochar treatments (Figure 1).

### 3.3. Soil physic-chemical properties

As shown in Table 2, the results of two-way ANOVA showed that biochar significantly affected soil pH, organic matter, alkeline-N, available P and available K (*p* < 0.001). AM fungi significantly affected alkeline-N and available P (*p* < 0.01). The addition of biochar significantly increased soil organic matter and available K content. Inoculation with AM fungi significantly increased soil alkeline-N content. While the combined application of biochar and AM fungi increased available P content, especially the combination of AM fungi and biochar prepared at 500°C (Table 3).

**Table 2:** Analysis of variance (*p*-values).

Source		Two-way ANOVA		
		AMF	BC	AMF × BC
Total Pb concentration and Pb fractions proportion	Total Pb concentration	<0.001^***^	0.010^*^	0.446
	Exchangeable (salt displaceable)	0.127	0.110	0.153
	Carbonates (acid extractable)	0.001^**^	<0.001^***^	0.017^*^
	Fe and Mn oxides (reducible)	0.001^***^	0.027^*^	0.022^*^
	Organic and sulfides (oxidisable)	0.081	0.031^*^	0.103
	Residual	0.942	<0.001^***^	0.003^**^
Soil physicochemical properties	pH	0.117	<0.001^***^	0.747
	Organic matter	0.099	<0.001^***^	0.372
	Alkeline-N	<0.001^***^	<0.001^***^	0.171
	Available P	0.002^**^	<0.001^***^	0.115
	Available K	0.487	<0.001^***^	0.256

**Table 3:** Soil physicochemical properties after application of AM fungi and biochar (mean ± SE).

Parameters treatments	pH	Organic matter (g/kg)	Alkeline-N (mg/kg)	Available P (mg/kg)	Available K (mg/kg)
NM0	7.88 ± 0.10^aB^	21.47 ± 0.23^cA^	41.45 ± 1.01^bB^	61.95 ± 1.47^bB^	168.00 ± 11.27^cA^
NM3	7.54 ± 0.08^bB^	45.90 ± 2.26^bA^	45.44 ± 1.01^aB^	100.97 ± 1.92^aB^	398.00 ± 17.32^aA^
NM4	7.72 ± 0.21^abB^	54.93 ± 6.18^aA^	39.90 ± 1.33^bB^	98.73 ± 8.64^aB^	330.00 ± 62.64^bA^
NM5	7.75 ± 0.01^abB^	52.86 ± 3.12^aA^	39.46 ± 1.38^bB^	99.21 ± 2.64^aB^	416.0 ± 6.93^aA^
AM0	8.02 ± 0.10^aA^	21.80 ± 0.38^cA^	45.45 ± 0.77^aA^	69.47 ± 0.73^cA^	167.67 ± 6.43^dA^
AM3	7.57 ± 0.07^cA^	45.44 ± 0.88^bA^	46.55 ± 1.33^aA^	106.73 ± 3.62^aA^	386.00 ± 29.60^bA^
AM4	7.79 ± 0.03^bA^	49.66 ± 4.04^aA^	43.23 ± 1.15^bA^	97.77 ± 1.47^bA^	326.00 ± 21.07^cA^
AM5	7.75 ± 0.17^bA^	49.78 ± 0.37^aA^	41.90 ± 0.67^bA^	109.45 ± 3.87^aA^	464.00 ± 13.86^aA^

NM0, no AM inoculation or biochar addition; NM3, no AM inoculation but 300°C biochar addition; NM4, no AM inoculation but 400°C biochar addition; NM5, no AM inoculation but 500°C biochar addition; AM3, introduced AM inoculation and 300°C biochar addition; AM4, introduced AM inoculation and 400°C biochar addition; AM5, introduced AM inoculation and 500°C biochar addition; AM0, introduced AM inoculation and no biochar addition. Different lower-case letters indicate significant differences among biochar treatments (Duncan’s comparisons, *p* < 0.05) and upper-case letters indicate significant differences between AM fungal inoculation treatments (NM vs AM, Tukey’s comparisons, *p* < 0.05).

### 3.4. Total Pb content and Pb speciation

**Figure 2:**
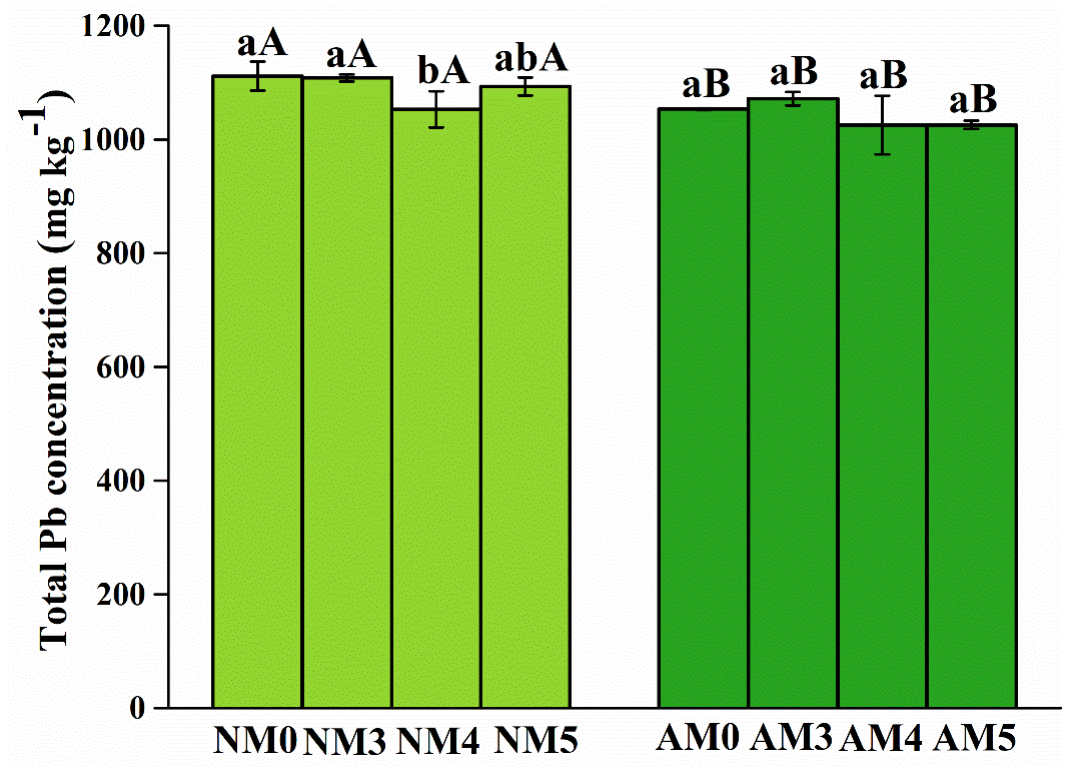
Total Pb concentration in soils after application of AM fungi and biochar prepared at different pyrolysis temperatures. NM0, no AM inoculation or biochar addition; NM3, no AM inoculation but 300°C biochar addition; NM4, no AM inoculation but 400°C biochar addition; NM5, no AM inoculation but 500°C biochar addition; AM3, introduced AM inoculation and 300°C biochar addition; AM4, introduced AM inoculation and 400°C biochar addition; AM5, introduced AM inoculation and 500°C biochar addition; AM0, introduced AM inoculation and no biochar addition. Different lower-case letters indicate significant differences among biochar treatments (Duncan’s comparisons, *p* < 0.05) and upper-case letters indicate significant differences between AM fungal inoculation treatments (NM vs. AM, Tukey’s comparisons, *p* < 0.05).

As shown in Table 2, the results of two-way ANOVA showed that AM fungi and biochar significantly affected the total Pb concentration and the acid extractable, reducible, oxidizable and residual fractions of Pb in soils (*p* < 0.05). It can be seen from Figure 2 that the difference of total Pb content in soil under different treatments was significant. The total Pb content in soil treated with AM4 and AM5 decreased significantly and was 1,025 mg kg^−1^.

**Figure 3:**
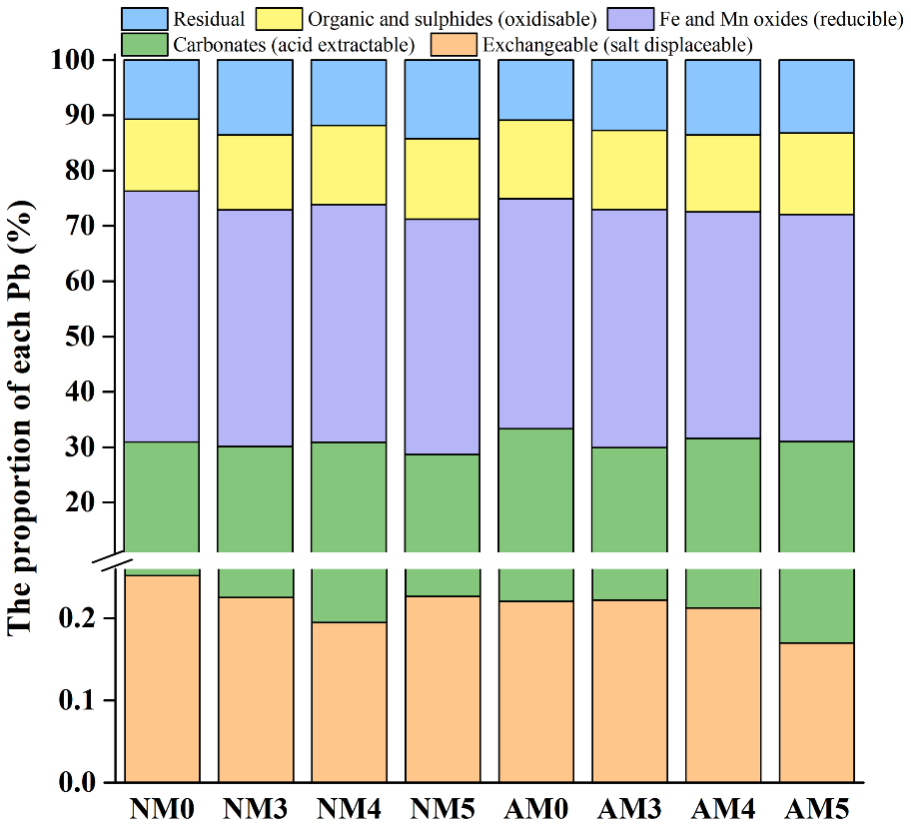
Distribution of different fractions of Pb in soils after application of AM fungi and biochar prepared at different pyrolysis temperatures.

The distribution of Pb in various fractions was clearly altered by the treatments with biochar and AM fungi. Different treatments had significant effects on the percentage of exchangeable, acid extractable, reducible, oxidizable and residual Pb fractions in soils (*p* < 0.05). In NM0 soil, Pb distributed mainly in acid extractable fractions (>30%) and reducible fractions (>45%). Compared to control soil (NM0), the proportion of exchangeable Pb and reducible Pb significantly decreased, respectively by 20 and 5.4%, while the proportions of oxidizable and residual Pb increased, respectively, by 9.3 and 10.8% under NM4 treatment. Similar differences were observed in AM5 with exchangeable Pb and reducible Pb decreased, respectively, by 32 and 9.5%, while oxidizable and residual Pb increased, respectively, by 13.2 and 23.5%. Under NM5 treatment, the proportion of acid extractable Pb and reducible Pb decreased significantly by 7.3 and 6.3%, respectively, while the proportion of oxidizable Pb and residual Pb increased by 12.33 and 32.9%, respectively. The content of Pb was found to decrease, respectively by 5.8, 5.2 and 9.8% in the reducible fraction while increase, respectively by 26.9, 19.1 and 26.6% in the residual fraction under the treatment of NM3, AM3 and AM4 (Figure 3 and Supplementary Table S1).

### 3.5. Microbial community diversity and composition

After quality filtering, a total of 1,731,285 bacterial sequences with an average length of 420 bases and 2,256,424 fungal quality sequences with an average length of 233 bases were obtained from 24 soil samples. The rarefaction curve has indicated that the sequencing effort was enough to represent bacterial and fungal diversity, since the curve has reached the platea (Supplementary Figure S3). A total of 53,107 bacterial and 3,449 fungal amplicon sequence variants (ASVs) were found. Different biochar and AM fungi treatments shared 1,173 bacterial and 237 fungal ASVs. More unique ASVs were found in AM4 and AM5 treated soils than that in the corresponding soils treated with NM4 and NM5 (Supplementary Figure S4).

The alpha diversity indices of either bacteria or fungi were not influenced by biochar, AM fungi and the interaction between bacteria and AM fungi (Supplementary Table S2).

**Figure 4:**
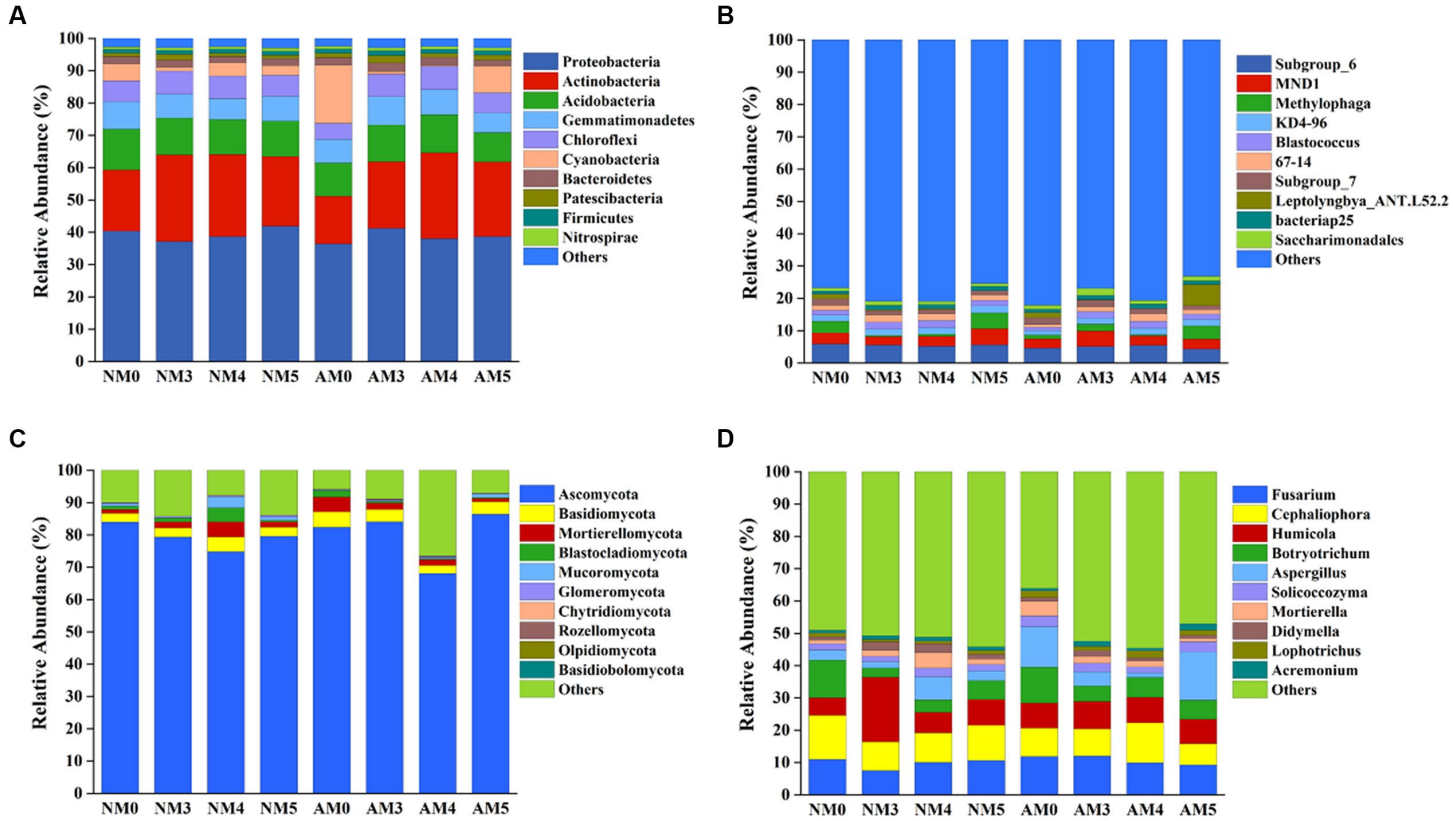
Changes in the microbial communities at the phylum and genus levels. **(A)** Bacteria phyla. **(B)** Bacteria genera. **(C)** Fungi phyla. **(D)** Fungi genera.

The relative abundances of the top 10 bacterial and fungal phyla and genera under different treatments were shown in Figure 4. The dominant bacterial phyla across all soil samples were Proteobacteria, Actinobacteria, and Acidobacteria (Figure 4A). The dominant fungal phyla across all soil samples were Ascomycota, Basidiomycota and Mortierellomycota (Figure 4C). Biochar, AM fungi, and their interactions significantly affected the relative abundances of some dominant bacteria and fungi at the phylum and genus levels (Supplementary Table S3), which were related to soil parameters such as pH, organic matter, available P and available K (Supplementary Table S4). More specifically, compared to NM0 treatment, NM5 and AM3 treatments exerted positive effects on the relative abundance of Proteobacteria. The application of biochar alone increased the abundance of Actinobacteria (Figure 4A). AM3 treatment increased the abundance of *Saccharimonadales* (Figure 4B and Supplementary Table S3). For the fungal community, the amendment of biochar and AM fungi decreased the abundance of Ascomycota, except for the slight increase in AM5 treatment (Figure 4C). The application of biochar alone decreased the abundance of *Botryotrichum*, but increased the abundance of *Acremonium* (Figure 4D and Supplementary Table S3).

**Figure 5:**
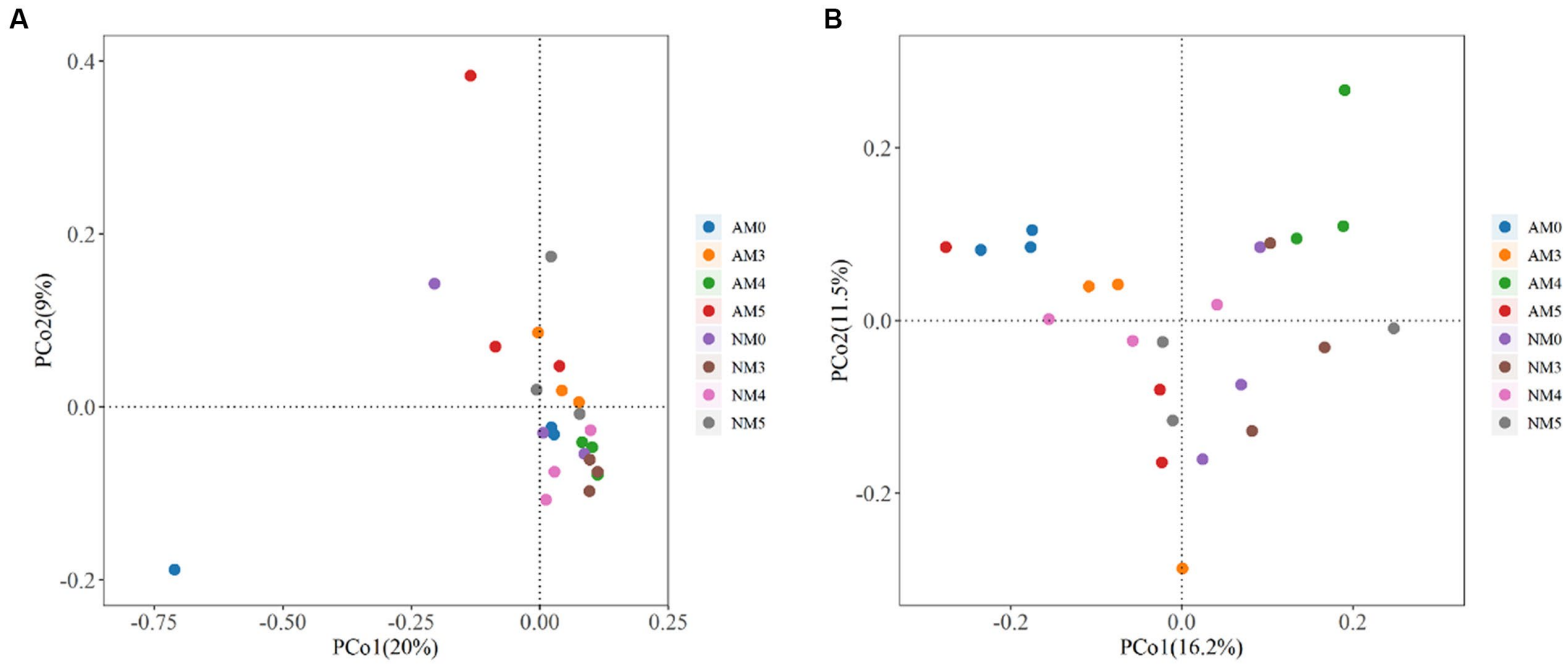
Principal coordinate analysis (PCoA) plots based on the Bray-Curtis distance showing the dissimilarity of microbial communities among different treatments. **(A)** The bacterial communities. **(B)** The fungal communities.

We further investigated the dissimilarity of microbial communities among different treatments through two dimensional principal coordinates analysis (PCoA) based on the Bray-Curtis distance (Figure 5). Adonis analysis indicated that biochar and AM fungi affected the composition of the bacterial community to some extent (Adonis: *R*^2^ = 0.379, *p* = 0.001). The PCoA components explained 20% (PCo1) and 9% (PCo2) of the total variance and confirmed a separation between the soils treated with NM0 and AM5, AM4, AM3, NM5 and NM3, while the soils treated with AM3, AM4, AM5 were separated from AM0. In addition, the soils treated with AM4 was markedly different from the soils treated with AM3 and AM5, but AM3 and AM5 shared a similar community structure (Figure 5A).

Similarly, the composition of fungal community was also affected by the treatments with biochar and AM fungi (Adonis: *R*^2^ = 0.393, *p* = 0.003). The PCoA components explained 16.2% (PCo1) and 11.5% (PCo2) of the total variance and confirmed a separation between the soils treated with NM0 and NM3, NM4, AM0, AM3, AM4 and AM5, while the soils treated with AM3, AM4, AM5 were segregated from AM0. Furthermore, the AM4 treated soil was markedly different from both AM3 and AM5 soils, but AM3 and AM5 shared a similar community structure. Similarly, the soil treated with NM4 was markedly different from the soils treated with NM3 and NM5, but NM3 and NM5 shared a similar community structure (Figure 5B).

**Figure 6:**
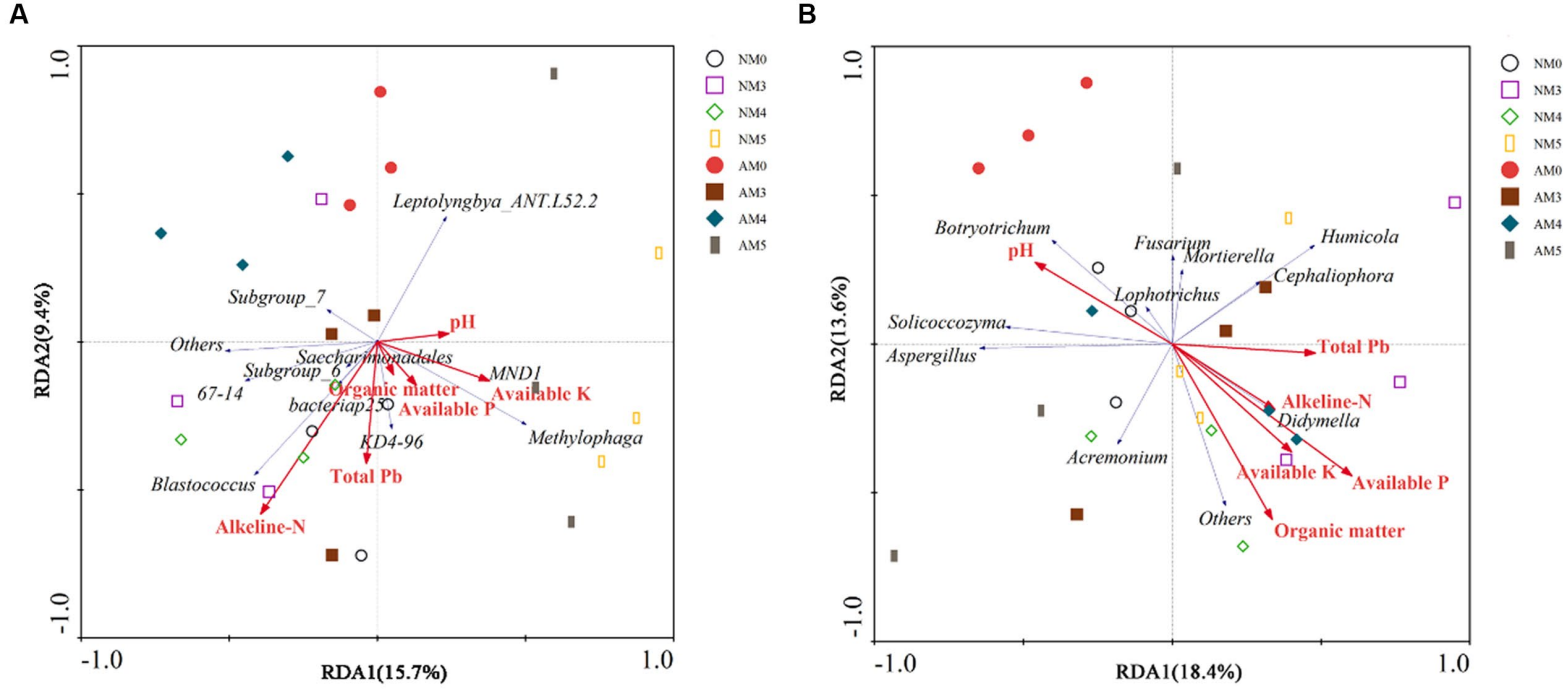
Redundancy analysis (RDA) ordination of the bacterial communities **(A)** and fungal communities **(B)** constrained by soil chemical properties. The red arrows display explanatory variables (soil chemical properties) and response variables (microbial community) are shown in blue arrows. The different graphical symbols show soil samples.

According to the results of DCA, the lengths of the DCA1 gradient were, respectively, 0.582 and 1.494 for the bacterial and fungal communities, shorter than 4.0. Therefore, RDA was selected to further investigate the effects of environmental factors on the microbial communities. The first axis accounted for 15.7% and the second axis explained only 9.4% of the total variation in bacterial communities. For RDA1, available K and pH were the main influential constraining variable correlated with bacterial communities. Alkeline-N was the most influential constraining variable for RDA2, along with total Pb (Figure 6A). In addition, RDA results showed that the first axis was associated with 18.4% of the total variation in the fungal communities, and the second axis accounted for 13.6% of the variability. The available P, pH, alkeline-N and total Pb mainly affected the distribution of fungal communities in the first axis, and organic matter and available K mainly affected the distribution of fungal communities in the second axis (Figure 6B). The results of Monte Carlo permutation test showed that available P (*F* = 2.63, *p* = 0.042) was the most influential constraining variable correlated with fungal communities.

### 3.6. Predicted bacterial function profiles

**Figure 7:**
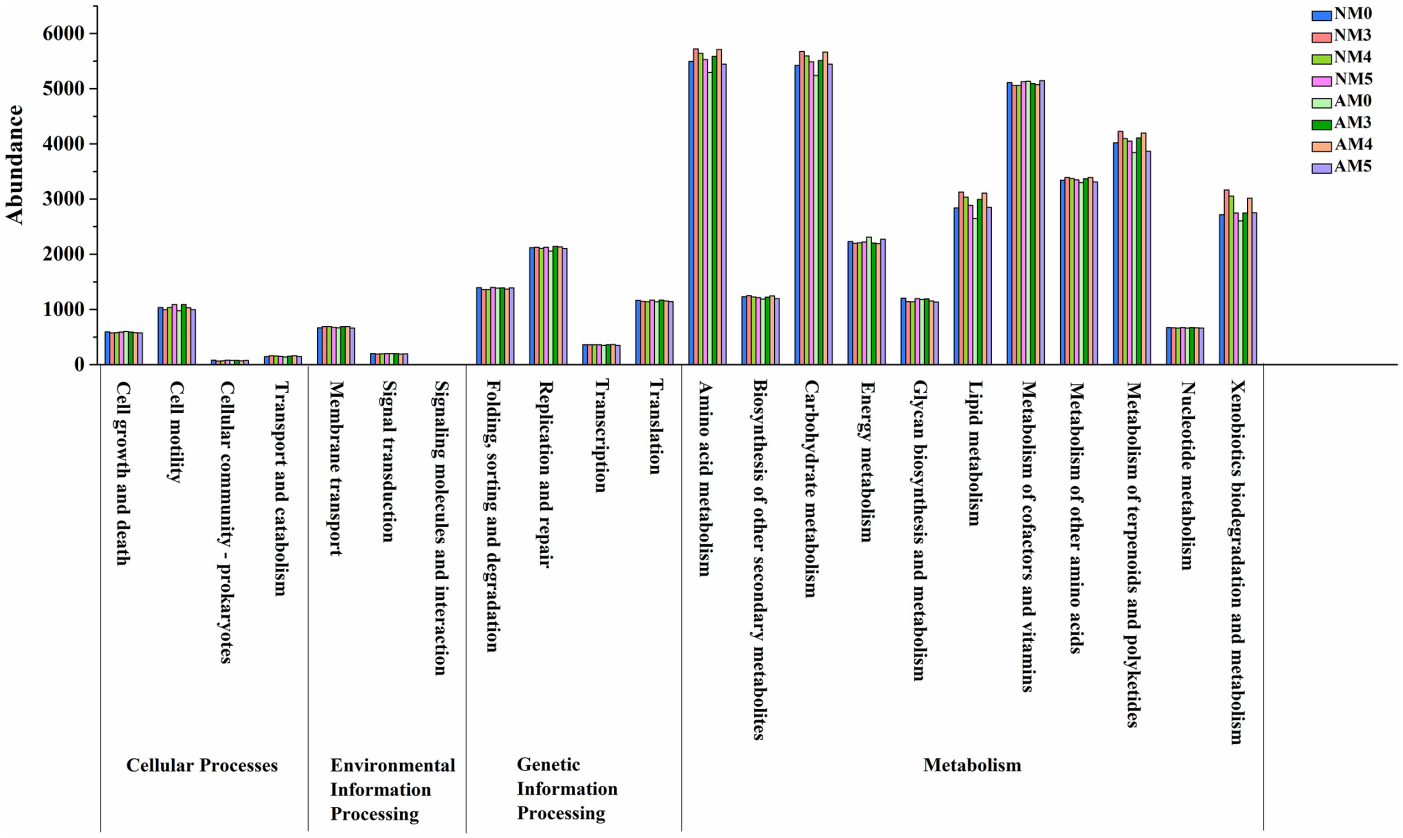
Variation of bacterial function profiles under different treatments analyzed by PICRUSt.

Bacterial functional profiles were predicted for different treatments using the PICRUSt software package. The functional gene abundances from the KEGG pathway hierarchy level 2 were analyzed. The results showed that the overall patterns of the metabolic pathways were similar in different treatments. But most of the functional gene abundances involved in amino acid, carbohydrate, lipid, terpenoids and polyketides metabolism, and xenobioties biodegradation and metabolism were obviously enriched by biochar amendment (Figure 7). It indicated that bacteria functions were potentially stimulated by biochar amendment, but little by inoculation with introduced AM fungi.

## 4. Discussion

### 4.1. Effects of biochar and AM fungi on Pb content and Pb speciation

In this study, the Pb content in AM4 or AM5 treated soils was the lowest, which indicated that the combined application of AM fungi and biochar prepared at 400°C or 500°C can effectively reduce the soil Pb content. But the effect of biochar prepared at 300°C was not obvious, which may be attributed to the difference in the surface characteristics of biochar prepared at different carbonization temperatures. Studies found that biochar with a larger specific surface area was more favorable to adsorb heavy metals in soil, and the properties of functional groups on the surface of biochar, such as volatile matter, oxygen content, and pH, also afffected its ability to adsorb heavy metals (Tang et al., 2013; Tomczyk et al., 2020).

In addition, we also found that the combined application of AM fungi and biochar prepared at 400°C or 500°C can significantly promote the transformation of Pb from the exchangeable state, carbonate bound state and iron manganese oxidation state with high bioavailability to residue state with low bioavailability, thus reducing its bioavailability. It has been widely proved that biochar can reduce Pb pollution in soil due to the porous structure and the high surface area consisting of numerous functional groups, which could provide enormous exchange sites and adsorb Pb^2+^ through electrostatic interaction, precipitation, cation exchange, and surface complexation (Tang et al., 2013; Paz-Ferreiro et al., 2014; El-Naggar et al., 2020; Naeem et al., 2021). Futhermore, the effects of AM fungi inoculation on the decrease of heavy metal bioavailability have also been confirmed by many studies (Aghababaei et al., 2014; Turan, 2021). On the one hand, AM fungi can dissolve heavy metals bound by carbonate and oxide and increase their accumulation in the residual fraction by stimulating microbial activity and acidifying the rhizosphere by releasing simple organic acids (Aghababaei et al., 2014; Hu et al., 2021; Turan, 2021). And, on the other hand, the glomalin-related soil protein produced by AM mycelia is also well known for its putative role in immobilizing heavy metals in soil (Vodnik et al., 2008; Wu et al., 2014). Therefore, the combination of biochar and AM fungi may achieve a better effect on stabilizing heavy metals in soil. Khan et al. (2020) proved the associative effects of biochar and AM fungi on the reduction of bioavailable Pb concentration in soil. Liu et al. (2018) found that combined biochar and AM fungi treatments showed the most pronounced effect on the conversion of soil Cd from the acid extractable and reducible fractions to the oxidizable and residual fractions. Turan (2021) reported similar results on the reduction of Ni by combining the two treatments. All of these results are consistent with our findings, indicating a synergistic positive effect between biochar and AM fungi.

### 4.2. Effects of biochar and AM fungi on the microbial community

Although changed microbial diversity was detected in biochar-amended soil by many studies (Xu et al., 2016; Cheng et al., 2018; Li X. et al., 2020; Wan et al., 2022). Our results indicated that the alpha diversity of bacteria and fungi did not differ significantly under the treatments of biochar and AM fungi. Similarly, Li S. et al. (2020) also found that the alpha diversity of bacterial and fungal hardly changed after 2 years of addition of biochar alone. The inconsistent research conclusions indicated that the effects of biochar on microbial diversity still largely depended on biochar application rates, biochar properties and soil types. Because the chemical properties (especially pH and nutrient content) and physical properties (such as pore size, pore volume, and specific surface area) of biochar play significant roles in determining the efficacy of biochar on microbial performance as biochar provides suitable habitats for microorganisms (Palansooriya et al., 2019). In addition, the reports on the impact of AM fungi on microbial diversity were also inconsistent. Some reports were consistent with our research findings, the presence of AM fungi did not significantly alter the total microbial diversity although it significantly modified the microbial community (Nuccio et al., 2013; Gui et al., 2017). But the relationships and influencing mechanisms between AM fungi and microbial diversity are still largely unknown.

In this study, the composition of the bacterial community was influenced by biochar combined with AM fungi at the levels of phylum and genus (Figures 4A,B). The biochar amendment obviously increased the relative abundance of Actinobacteria and *Blastococcus*. The treatment with AM3 increased the relative abundance of Proteobacteria. Proteobacteria and Actinobacteria were the considerably highest abundant phylum in all treatments. These two phyla with the vast majority of heavy metal resistance genes have strong adaptability and are the dominant microbes in many soils under heavy metal stress (Li S. et al., 2020; Yan et al., 2020; Li M. et al., 2022). Therefore, the increased abundance of Proteobacteria and Actinobacteria could be due to the resistant abilities of microorganisms to Pb. Additionally, the application of biochar alone significantly increased the abundance of *Blastococcus*, which belongs to the family Geodermatophilaceae and also has high resistance to heavy metal stress (Chouaia et al., 2012). For the fungi, the relative abundance of phyla and genera also showed different distributions among the treatments (Figures 4C,D). The amendment of biochar and AM fungi decreased the abundance of Ascomycota, except for a slight increase in AM5 treatment. Ascomycota has been reported to be the key decomposer of crop residue in the agricultural soil (Lin et al., 2019).

Furthermore, in our study, distinct group separations were observed between the bacterial and fungal communities of different treatments (Figure 5), indicating that the bacterial and fungal communities changed significantly with the application of biochar and AM fungi. In terms of the bacterial community, it should be noted that we found that the soils treated with AM3, AM4 and AM5 were all separated from AM0. However, there was no significant separation between AM0 and NM0, AM4 and NM4, and AM5 and NM5 (Figure 5A), which indicated that biochar amendment was responsible for the changes in the bacterial community. The reason may be that biochar improved nutrient retention in soil micropores by significantly increasing soil organic matter and available K content (Table 3), which affected the availability of nutrient elements by microorganisms (Cheng et al., 2018). With regard to fungi, the soil treated with AM3, AM4 and AM5 were also separated from AM0. But the difference was that there were distinct separations between AM0 and NM0, AM3 and NM3, AM4 and NM4 and AM5 and NM5 (Figure 5B), which meant that biochar and AM fungi were responsible for the change of the fungal community. In particular, whether bacterial or fungal community, the treated soil with AM4 was markedly different from that with AM3 or AM5, but both AM3 and AM5 treated soils shared a similar community structure. This may be due to the obvious decrease in available P and available K contents of the soil caused by AM4 treatment, compared to AM3 and AM5 (Table 3).

### 4.3. Correlations with environmental factors and microbial community structures

In this study, RDA analysis confirmed that the application of AM fungi and biochar changed microbial communities by influencing the environmental parameters in Pb contaminated soil. The results showed that pH, alkeline-N and available K were the important parameters affecting the bacterial and fungal communities. Similar findings were also concluded in other studies (Palansooriya et al., 2019; Li S. et al., 2020; Yang et al., 2021). The alkeline-N and available K were the main nutrients in soil, which had a direct impact on the growth and metabolic activities of soil microorganisms. Soil pH could limit enzyme activities and enhance the resistance of specific microflora to environmental stress, thereby greatly affecting soil chemical processes and altering microbial communities (Sun et al., 2020). Furthermore, available P was explored as an important environmental parameter that shaped the fungal community structure, which may be attributed to the increased abundance of some phosphate solubilizing microbial species under the combination of biochar and AM fungi (Li S. et al., 2022).

### 4.4. Effects of biochar and AM fungi on bacterial function

We analyzed whether the bacterial community in the AM fungi and biochar treated soils produce a distinct functional profile using Pircust. Our results indicated that the biochar treated soils were functionally distinct. 6 functional groups obviously exhibited higher abundance in biochar-amended soils. These functions were involved in carbon, nitrogen, and phosphorous related metabolism or cycling, and xenobioties biodegradation and metabolism. It can be explained by that biochar can improve nutrient retention capability and act as a slow-release fertilizer, which can release nutrients at different rates to bring long-term benefits for microbial growth (Lu et al., 2021). Therefore, the enhancement of these traits through biochar amendment may reflect a more sustainable soil microbial community.

## 5. Conclusion

This study demonstrated that introduced AM fungi combined with biochar prepared at 400°C or 500°C significantly improved soil nutrients, and promoted the transformation of Pb form to a more stable state with low bioavailability under field conditions, which suggested that the combined application of introduced AM fungi and biochar prepared at 400°C or 500°C was an effective strategy for Pb remediation in agricultural soils. The application of biochar and introduced AM fungi also had significant impacts on native microbial community compositions. The changes of soil microbial community compositions were indirectly driven by alternation of soil characteristics which were highly correlated with biochar addition. In addition, the bacterial metabolic functions involved in carbon, nitrogen, and phosphorous related metabolism and xenobioties biodegradation were significantly enriched in biochar-amended soils, which suggested that biochar addition could effectively improve the soil micro- environment during the process of Pb remediation and its effect was superior to the introduced AM fungi.

## Data availability statement

The datasets presented in this study can be found in online repositories. The names of the repository/repositories and accession number(s) can be found in the article/Supplementary material.

## Author contributions

XC: Formal analysis, Methodology, Writing – original draft, Writing – review & editing. LT: Investigation, Resources, Writing – original draft. KW: Data curation, Writing – original draft. YM: Data curation, Writing – original draft. QT: Data curation, Validation, Writing – original draft. GL: Funding acquisition, Software, Visualization, Writing – original draft. YZ: Writing – review & editing.

## Footnotes

^1^ https://docs.qiime2.org/2019.4/tutorials/
