# Supplementary material for: Combined contribution of biochar and introduced AM fungi on lead stability and microbial community in polluted agricultural soil
Source: Front Microbiol. 2023 Nov 15;14:1284321. doi: 10.3389/fmicb.2023.1284321 (PMC10684681; doi:10.3389/fmicb.2023.1284321)
Supplement: Supplementary file 1 [file Data_Sheet_1.pdf]

Supplementary Materials

Combined contribution of biochar and introduced AM fungi on lead stability and microbial community in polluted agricultural soil

Xuedong Chen[1,*], Lin Tang[1], Kongyang Wu[1], Yifan Mo[1], Qian Tang[1], Gaojie Li[2,*], Ying Zhu[1]

[1] *College of Life Science, Luoyang Normal University, Luoyang 471934, Henan,China*

[2] *School of Physics and Engineering, Henan University of Science and Technology, Luoyang 471023, Henan, China*

The corresponding author:

Xuedong Chen

Email: chenxuedong1224@163.com

ORCID: 0000-0001-7041-5015

Gaojie Li

Email: ligjie2019@haust.edu.cn

Table S1 Individual fractions of Pb in soils after application of AM fungi and biochar prepared at different pyrolysis temperatures (mean±SE)

Treatments	Pb concentration of individual fractions (mg kg^{-1})				
	Exchangeable	Acid extractable	Reducible	Oxidisable	Residual
NM0	2.80 ± 0.06^{aA}	340.58 ± 18.54^{aA}	503.68 ± 4.84^{aA}	144.17 ± 9.72^{aA}	118.85 ± 0.93^{bA}
NM3	2.45 ± 0.56^{abA}	324.75 ± 3.15^{abA}	464.05 ± 12.21^{bA}	147.03 ± 5.66^{aA}	147.53 ± 3.23^{aA}
NM4	2.02 ± 0.15^{bA}	318.45 ± 4.87^{abA}	445.00 ± 13.89^{cA}	148.36 ± 2.57^{aA}	123.05 ± 3.79^{bA}
NM5	2.47 ± 0.05^{abA}	309.40 ± 11.83^{bA}	462.58 ± 2.37^{bcA}	158.56 ± 10.07^{aA}	154.70 ± 6.72^{aA}
AM0	2.22 ± 0.22^{abB}	333.39 ± 13.52^{aA}	419.30 ± 8.09^{bB}	142.85 ± 3.32^{aA}	110.00 ± 1.42^{bA}
AM3	2.30 ± 0.53^{aB}	307.19 ± 7.04^{bA}	444.97 ± 10.75^{aB}	148.08 ± 5.77^{aA}	131.99 ± 3.50^{aA}
AM4	2.11 ± 0.02^{abB}	311.33 ± 11.92^{bA}	406.30 ± 4.06^{bB}	138.19 ± 8.54^{aA}	134.75 ± 11.30^{aA}
AM5	1.70 ± 0.03^{bB}	309.34 ± 4.50^{bA}	411.69 ± 12.99^{bB}	147.33 ± 3.19^{aA}	132.67 ± 10.71^{aA}

Note: NM0: no AM inoculation or biochar addition; NM3: no AM inoculation but 300°C biochar addition; NM4: no AM inoculation but 400°C biochar addition; NM5: no AM inoculation but 500°C biochar addition, AM3: introduced AM inoculation and 300°C biochar addition; AM4: introduced AM inoculation and 400°C biochar addition, AM5: introduced AM inoculation and 500°C biochar addition; AM0: introduced AM inoculation and no biochar addition. Different lower-case letters indicate significant differences among biochar treatments (Duncan's comparisons, $P < 0.05$) and upper-case letters indicate significant differences between AM fungal inoculation treatments (NM vs AM, Tukey's comparisons, $P < 0.05$).

Table S2 Community diversity indices of bacteria and fungi based on the 16S and ITS rRNA genes (mean ± SE)

		Treatments								Two-way ANOVA		
	Variables	NM0	NM3	NM4	NM5	AM0	AM3	AM4	AM5	AMF	BC	AMF × BC
Bacteria	Chao1	6342.74 ± 964.74	6194.07 ± 693.08	5815.17 ± 444.98	6668.26 ± 357.63	5217.62 ± 2487.40	6723.61 ± 287.98	6751.69 ± 572.52	6015.33 ± 1154.46	0.864	0.724	0.352
	Observed_species	5568.30 ± 750.76	5614.70 ± 462.21	5205.87 ± 370.52	5802.67 ± 299.07	4710.23 ± 2131.67	6037.03 ± 207.40	5947.47 ± 463.64	5329.47 ± 802.69	0.911	0.622	0.401
	Shannon	10.63 ± 0.77	11.12 ± 0.04	10.86 ± 0.18	10.78 ± 0.29	9.58 ± 2.58	11.10 ± 0.07	11.18 ± 0.08	10.14 ± 0.87	0.411	0.301	0.648
	Simpson	0.995 ± 0.006	0.999 ± 0.000	0.998 ± 0.001	0.995 ± 0.004	0.974 ± 0.042	0.999 ± 0.001	0.999 ± 0.000	0.984 ± 0.016	0.254	0.374	0.630
Fungi	Chao1	453.48 ± 51.61	529.28 ± 41.83	470.02 ± 57.81	506.63 ± 45.68	492.10 ± 57.25	524.79 ± 26.22	515.68 ± 16.19	516.41 ± 51.43	0.248	0.233	0.752
	Observed_species	452.40 ± 50.82	528.00 ± 42.34	469.70 ± 57.42	505.37 ± 45.45	491.60 ± 57.22	524.33 ± 26.06	515.20 ± 16.30	515.87 ± 50.88	0.236	0.232	0.758
	Shannon	5.79 ± 0.11	5.72 ± 0.92	6.23 ± 0.14	6.03 ± 0.53	6.06 ± 0.46	5.92 ± 1.11	5.90 ± 0.27	5.89 ± 0.32	0.999	0.914	0.797
	Simpson	0.95 ± 0.02	0.93 ± 0.05	0.97 ± 0.01	0.95 ± 0.02	0.96 ± 0.01	0.91 ± 0.11	0.95 ± 0.02	0.93±0.04	0.574	0.537	0.914

Table S3 Relative abundances (%) of dominant genera of bacteria and fungi (mean ± SE)

	Treatments									Two-way ANOVA		
	Genera	NM0	NM3	NM4	NM5	AM0	AM3	AM4	AM5	AMF	BC	AMF×BC
Bacteria	*Subgroup_6*	5.84±1.71	5.51±0.23	5.19±0.74	5.57±0.42	4.53±3.21	5.06±1.05	5.43±0.70	4.31±1.68	0.28	0.973	0.784
	MND1	3.45±0.34^{bA}	2.63±0.32^{bA}	3.06±1.22^{bA}	5.07±0.61^{aA}	2.90±2.11^{aA}	4.79±1.78^{aA}	2.95±0.28^{aA}	3.08±1.08^{aA}	0.799	0.394	0.049*
	Methylophaga	3.56±5.72	0.32±0.41	0.65±0.93	4.84±5.62	1.23±1.08	2.17±2.32	0.32±0.47	4.13±5.03	0.792	0.254	0.779
	KD4-96	2.02±0.36	2.00±0.07	1.97±0.28	2.19±0.28	1.33±0.88	1.92±0.20	2.06±0.23	1.94±0.54	0.197	0.418	0.441
	Blastococcus	1.42±0.11^{cA}	2.20±0.36^{abA}	2.29±0.35^{aA}	1.67±0.23^{bcA}	1.02±0.64^{bA}	1.95±0.50^{aA}	2.12±0.11^{aA}	1.66±0.27^{abA}	0.183	0.001**	0.822
	67-14	1.54±0.57^{aA}	2.18±0.11^{aA}	2.02±0.35^{aA}	1.70±0.09^{aA}	0.95±0.65^{bA}	1.57±0.82^{bA}	2.33±0.27^{aA}	1.37±0.29^{bA}	0.056	0.002*	0.141
	Subgroup_7	2.01±0.70	1.35±0.12	1.31±0.20	1.31±0.05	2.04±1.28	2.04±0.68	1.61±0.27	1.24±0.69	0.376	0.235	0.741
	Leptolyngbya_ANT.L52.2	1.49±1.34	0.26±0.27	0.25±0.39	0.06±0.02	1.62±2.26	0.09±0.14	0.02±0.01	6.57±11.30	0.366	0.511	0.431
	bacteriap25	0.96±0.27	1.37±0.15	1.37±0.37	1.32±0.17	1.03±0.73	1.37±0.13	1.41±0.06	1.13±0.29	0.87	0.19	0.904
	Saccharimonadales	0.95±0.26^{aB}	1.30±0.30^{aB}	0.91±0.28^{aB}	0.88±0.28^{aB}	1.19±0.14^{bA}	2.12±0.56^{aA}	1.03±0.04^{bA}	1.40±0.21^{bA}	0.003*	0.002*	0.212
Fungi	*Fusarium*	10.91±2.63	7.52±0.91	10.05±4.20	10.62±2.00	11.88±1.82	12.03±6.41	9.93±3.25	9.21±1.89	0.475	0.817	0.471
	Cephaliophora	13.72±1.24	8.81±3.31	9.10±1.94	10.89±0.36	8.76±1.41	8.34±6.44	12.35±0.60	6.61±1.23	0.173	0.27	0.073
	Humicola	5.44±0.31	20.06±23.02	6.37±1.20	8.01±2.68	7.78±1.71	8.53±2.40	7.87±3.71	7.56±3.60	0.564	0.385	0.478
	Botryotrichum	11.60±5.55^{aA}	2.94±0.41^{bA}	3.83±1.03^{bA}	5.85±2.71^{abA}	11.07±8.73^{abA}	4.81±0.86^{abA}	6.28±3.78^{abA}	5.92±3.31^{abA}	0.582	0.034*	0.915
	Aspergillus	3.18±1.52	1.84±0.39	7.18±8.13	3.00±2.01	12.52±5.57	4.36±3.57	1.33±0.74	14.98±16.03	0.127	0.411	0.149
	Solicoccozyma	1.90±0.59	1.73±0.65	2.79±1.18	2.02±0.70	3.38±1.48	2.70±0.90	1.88±0.71	3.12±1.55	0.138	0.883	0.227
	Mortierella	1.12±0.29	1.90±0.37	4.70±4.33	1.64±0.51	4.55±4.67	2.05±1.17	1.78±0.48	1.05±0.29	0.987	0.503	0.164
	Didymella	1.03±0.31	2.52±1.47	2.65±1.02	1.49±0.28	1.17±0.51	1.84±0.81	1.03±0.52	0.92±0.24	0.042*	0.074	0.293
	Lophotrichus	1.17±0.51	0.76±0.41	0.90±0.21	1.22±0.40	2.04±0.72	1.32±0.96	2.17±2.46	1.63±1.41	0.109	0.824	0.913
	Acremonium	0.89±0.55^{aA}	1.14±0.46^{aA}	1.25±0.25^{aA}	1.10±0.56^{aA}	0.78±0.20^{bA}	1.57±0.49^{aA}	0.81±0.28^{bA}	1.97±0.17^{aA}	0.264	0.032*	0.055

Note: NM0: no AM inoculation or biochar addition; NM3: no AM inoculation but 300℃ biochar addition; NM4: no AM inoculation but 400℃ biochar addition; NM5: no AM inoculation but 500℃ biochar addition, AM3: introduced AM inoculation and 300℃ biochar addition; AM4: introduced AM inoculation and 400℃ biochar addition, AM5: introduced AM inoculation and 500℃

biochar addition; AM0: introduced AM inoculation and no biochar addition. Different lower-case letters indicate significant differences among biochar treatments (Duncan's comparisons, $P < 0.05$) and upper-case letters indicate significant differences between AM fungal inoculation treatments (NM vs AM, Tukey's comparisons, $P < 0.05$). *: $P < 0.05$; **: $P < 0.01$.

Table S4 Person correlation of soil parameters with the dominant bacteria and fungi at the genus level.

	Genera	pH	Organic matter	Alkeline-N	Available P	Available K	Total Pb
Bacteria	*Subgroup_6*	-0.009	-0.080	0.244	0.025	-0.032	0.172
	MND1	-0.091	0.063	0.087	0.199	0.285	0.177
	Methylophaga	0.193	0.011	-0.076	0.030	0.152	0.079
	KD4-96	-0.221	0.364	0.135	0.419*	0.379	0.113
	Blastococcus	-0.511*	0.631**	0.264	0.566**	0.418*	-0.073
	67-14	-0.329	0.511*	0.148	0.519**	0.300	0.050
	Subgroup_7	0.082	-0.406*	0.183	-0.375	-0.360	0.022
	Leptolyngbya_ANT.L52.2	0.085	-0.029	-0.361	-0.038	0.026	-0.197
	bacteriap25	-0.426*	0.369	0.159	0.387	0.351	-0.136
	Saccharimonadales	-0.517**	0.116	0.199	0.169	0.265	-0.041
Fungi	*Fusarium*	0.126	-0.242	-0.038	-0.179	-0.128	0.170
	Cephaliophora	0.391	-0.209	-0.025	-0.140	-0.287	0.229
	Humicola	-0.311	0.075	0.096	0.260	0.225	0.139
	Botryotrichum	0.597**	-0.556**	-0.123	-0.591**	-0.546**	-0.032
	Aspergillus	0.116	-0.072	-0.257	-0.251	-0.089	-0.410*
	Solicoccozyma	0.011	-0.103	-0.164	-0.244	-0.105	-0.445*
	Mortierella	0.055	-0.149	-0.101	-0.165	-0.178	-0.043
	Didymella	-0.301	0.158	0.416*	0.283	0.183	0.166
	Lophotrichus	0.191	0.110	-0.252	-0.208	-0.198	-0.024
	Acremonium	-0.271	0.356	-0.015	0.322	0.423*	-0.178

Note: *: $P < 0.05$; **: $P < 0.01$.

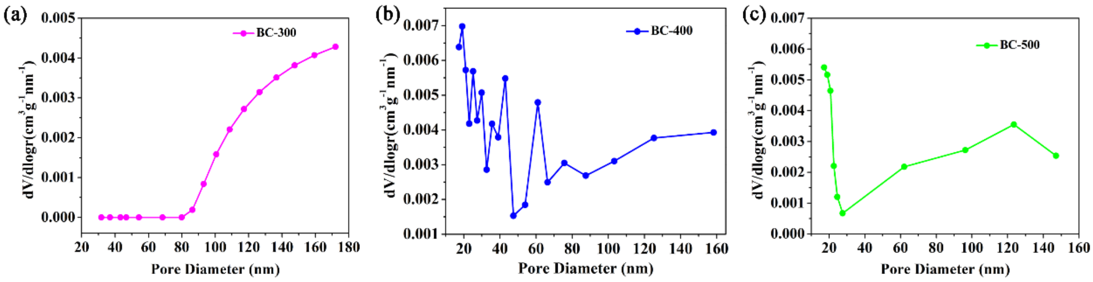

Figure S1 Pore size distribution of biochar prepared at different pyrolysis temperatures. (a):BC300; (b):BC400; (c):BC500

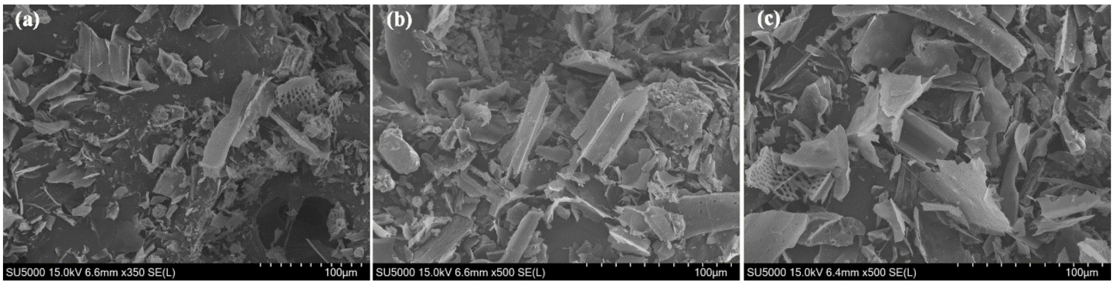

Figure S2 SEM images of biochar prepared at different pyrolysis temperatures. (a) BC-300, 100 μm; (b) BC-400, 100 μm; (c) BC-500, 100 μm

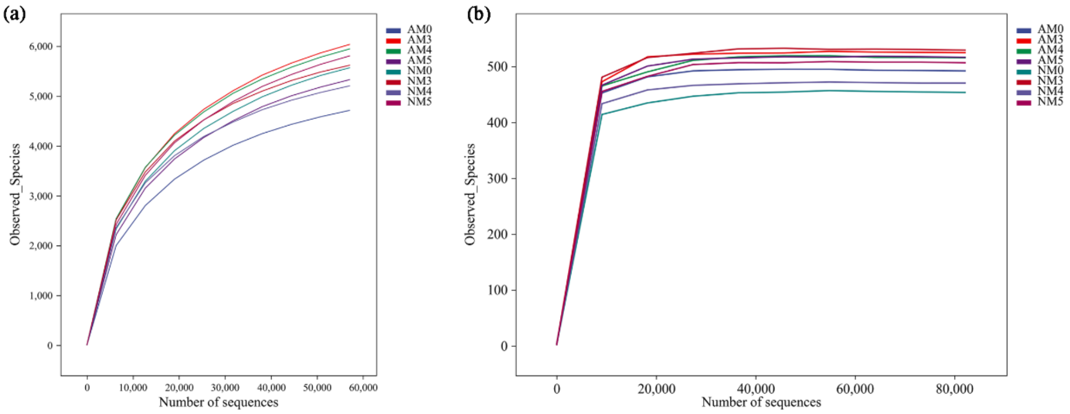

Figure S3 Rarefaction curves. (a): Bacteria; (b) Fungi.

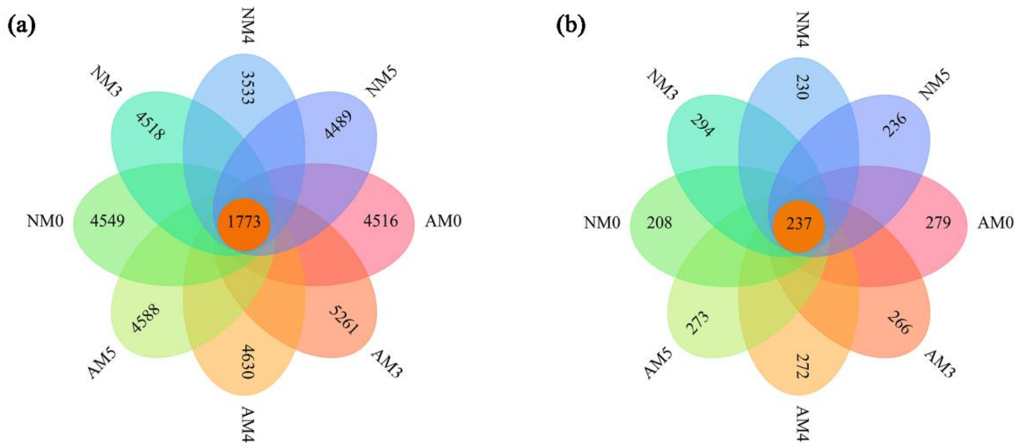

Figure S4 Venn diagrams of shared and unique ASVs among different treatments. (a): Bacteria; (b) Fungi.
